# Anti-Integrin αvβ6 Autoantibodies Predict Response and Treatment Persistence to Advanced Therapies in Ulcerative Colitis

**DOI:** 10.14309/ctg.0000000000000990

**Published:** 2026-02-03

**Authors:** Shunsuke Shibui, Kunio Asonuma, Satoshi Kuronuma, Shinji Okabayashi, Akira Nogami, Moeko Komatsu, Kanade Serizawa, Satoko Umeda, Shintaro Sagami, Galia Berman, Osamu Takeuchi, Masaru Nakano, Toshifumi Hibi, Nitsan Maharshak, Shin Maeda, Taku Kobayashi

**Affiliations:** 1 Center for Advanced IBD Research and Treatment, Kitasato University Kitasato Institute Hospital, Tokyo, Japan;; 2 Department of Gastroenterology, Yokohama City University, Graduate School of Medicine, Yokohama, Kanagawa, Japan;; 3 Biomedical Laboratory, Department of Research, Kitasato University, Kitasato Institute Hospital, Tokyo, Japan;; 4 Department of Healthcare Epidemiology, Graduate School of Medicine and Public Health, Kyoto University, Kyoto, Japan;; 5 Department of Gastroenterology, Kitasato University, Kitasato Institute Hospital, Tokyo, Japan;; 6 Department of Gastroenterology and Liver Diseases, Tel Aviv Medical Center, Affiliated with the Faculty of Medicine and Health Sciences, Tel Aviv University, Tel Aviv, Israel.

**Keywords:** ulcerative colitis, anti-integrin αvβ6 autoantibodies, advanced therapies, treatment outcome, biomarker

## Abstract

**INTRODUCTION:**

Anti-integrin αvβ6 (anti-αvβ6) autoantibodies serve as a diagnostic biomarker and are associated with poor prognosis in ulcerative colitis (UC). We aimed to investigate whether anti-αvβ6 autoantibody levels predict treatment outcomes of advanced therapies in patients with moderately to severely active UC.

**METHODS:**

Anti-αvβ6 autoantibody levels were measured using prospectively collected serum samples at the initiation of advanced therapies. The primary outcome was treatment persistence up to 1 year; secondary outcomes included clinical remission rates at weeks 2, 6, 14, 24, and 48, comparing low-level and high-level groups stratified by an optimal cutoff from receiver operating characteristic analysis.

**RESULTS:**

A total of 144 patients were analyzed (121 [84.0%] with extensive colitis and 87 [60.4%] with prior exposure to advanced therapies). The median observation period was 10 months, and treatment discontinuation occurred in 70 patients (48.6%). Treatment persistence was significantly higher in the low-level group (log-rank test, *P* = 0.002), and multivariable Cox analysis identified low antibody levels as the only independent predictor (hazard ratio, 1.90; 95% CI, 1.09–3.32). Clinical remission rates were consistently higher in the low-level group throughout all time points, with the greatest difference at week 6 (47.5% vs 20.0%; χ^2^ test, *P* = 0.003). Low antibody levels remained an independent predictor of remission at all time points.

**DISCUSSION:**

Anti-αvβ6 autoantibodies predicted both treatment persistence and clinical remission after advanced therapies, highlighting their potential as a predictive biomarker in patients with active UC.

## INTRODUCTION

The introduction of advanced therapies (ADTs), including biologics and small molecules, has significantly expanded treatment options for patients with ulcerative colitis (UC) (1). Despite these advances, approximately half of patients fail to respond to initial therapy (2), and even among responders, long-term maintenance is often unsuccessful (3). Therefore, predicting both short-term and long-term outcomes before initiating ADT is crucial for selecting the most appropriate treatment. However, implementing risk stratification prior to advanced therapy initiation remains a clinical challenge (1).

Anti-integrin αvβ6 (anti-αvβ6) autoantibodies have been reported as an accurate diagnostic biomarker for UC (4–7). Interestingly, recent studies have also linked them to adverse UC-related outcomes (8,9), suggesting their potential role as a prognostic biomarker. However, these studies assessed anti-αvβ6 autoantibody levels measured at diagnosis, leaving it unclear whether they predict poor treatment outcomes in patients initiating ADT. Their association with other poor prognostic factors also warrants further investigation. To address these questions, we conducted an observational study to clarify the association between anti-αvβ6 autoantibody levels and the treatment outcomes of ADT.

## METHODS

### Study design and participants

This was an observational study using baseline serum samples from a prospectively collected biobank cohort at Kitasato University Kitasato Institute Hospital (UMIN: 000053126, No. 19010) and Tel Aviv Medical Center (IRB: TLV-0162-19, NIH: NCT0499808; IRB: TLV-0268-19, NIH: NCT04912999). Patients with moderately to severely active UC who initiated ADT between June 1, 2017, and May 1, 2024, were included. ADT consisted of antitumor necrosis factor-α (TNF-α) agents, vedolizumab, interleukin (IL) -12/23 or IL-23 inhibitors, and Janus kinase (JAK) inhibitors.

The diagnosis of UC was based on clinical, endoscopic, and histologic criteria (10). The severity of UC was assessed using the modified Mayo score (11). Patients had to have a modified Mayo score between 4 and 9, with an endoscopic subscore ranging from 2 to 3 (11). Exclusion criteria were (i) age younger than 18 years; (ii) no colonoscopy within 6 months before enrolment; (iii) pregnancy; (iv) history of colectomy; and (v) missing baseline serum samples.

### Measurement

Serum samples collected at the time of ADT initiation were used to measure anti-αvβ6 autoantibody levels. Samples from Tel Aviv Medical Center were transported to Kitasato University Kitasato Institute Hospital for analysis. IgG antibodies against αvβ6 integrin were quantified using the Anti-Integrin αvβ6 ELISA kit (MBL, Tokyo, Japan), following the manufacturer's instructions. Absorbance was recorded at a primary wavelength of 450 nm and a reference wavelength of 620 nm using a microplate reader (MULTISKAN FC, Thermo Scientific).

### Outcomes

The primary outcome was treatment persistence in the low– and high–anti-αvβ6 autoantibody level groups. Secondary outcomes were clinical remission rates at weeks 2, 6, 14, 24, and 48 in the same groups. Treatment persistence was defined as continuation of ADT without switching to another ADT or undergoing UC-related surgery. The observation period was up to 1 year. Dose escalation or interval shortening of the advanced therapy, as well as the addition or dose escalation of concomitant nonadvanced therapies (e.g., 5-aminosalicylic acid, topical agents, or systemic corticosteroids), were considered continuation of treatment, provided that the initially prescribed advanced therapy was maintained. Clinical remission was defined as a patient-reported outcome-2 (PRO-2) score ≤1 with no rectal bleeding subscore (12). Patients who switched to another advanced therapy or underwent surgery were classified as nonclinical remission.

### Baseline clinical and laboratory variables

Data were collected on age, sex, disease duration, disease extent, smoking status at the time of enrolment, history of ADT use, prospectively collected PRO-2, Mayo endoscopic subscore (MES) (13), the modified Mayo score, laboratory tests (C-reactive protein [CRP], leucine-rich alpha-2 glycoprotein [LRG], fecal calprotectin), and concomitant medications through medical chart review. Laboratory tests were measured on the day of inclusion.

Disease extent was classified as proctitis, left-sided colitis, or extensive colitis (14). MES was defined based on the most recent colonoscopy performed within 6 months before inclusion. The modified Mayo score is the sum of the stool frequency, rectal bleeding, and endoscopic subscores, with a maximum total of 9 (15). A score of 0–3, 4–6, and ≥7 indicates mild, moderate, and severe disease, respectively (11).

### Statistical analysis

Continuous variables were expressed as mean ± SD or median (IQR) and categorical variables as proportions. The relationship between anti-αvβ6 autoantibody levels and age, sex, disease duration, disease extent, smoking history, history of ADT use, PRO-2, MES, the modified Mayo score severity, CRP, LRG, and fecal calprotectin was assessed using the χ^2^ test and the Mann–Whitney *U* test. Associations between anti-αvβ6 autoantibody levels and age, CRP, and LRG were also assessed using Spearman correlation coefficient. Receiver operating characteristic (ROC) curve analysis was performed to evaluate the predictive performance of anti-αvβ6 autoantibody levels for treatment persistence. The optimal cutoff value was determined using the Youden index on the ROC curve. Treatment persistence was analyzed using the Kaplan–Meier method with the log-rank test, and hazard ratios (HRs) were estimated using a Cox proportional hazards model. Censoring was defined as treatment discontinuation because of drug intolerance, transfer to another hospital, interruption of visits for more than 6 months, death unrelated to UC, surgery for UC-related colorectal cancer, or the date of last observation. Clinical remission rates were compared using the χ^2^ test, and odds ratios were estimated using logistic regression analysis. Patients who discontinued treatment because of intolerance, transfer, interrupted visits, unrelated death, or surgery for UC-related colorectal cancer were excluded from the analysis of remission rates. In all regression models, multivariable analyses were conducted to adjust for potential confounders, including disease duration, disease extent, history of ADT use, the modified Mayo score, and CRP. Missing values were reported in the tables and excluded from statistical analyses. All statistical analyses were performed using JMP version 17.0 (SAS Institute, Cary, NC). A *P*-value < 0.05 was considered statistically significant, and 95% CIs were calculated where appropriate.

### Ethics

The study protocol was reviewed and approved by the Research Ethics Committee of Kitasato University Kitasato Institute Hospital and Tel Aviv Medical Center (UMIN000053098, No. 23030). The study was conducted in accordance with the Declaration of Helsinki and Good Clinical Practice guidelines. Patient consent was obtained using an opt-out approach at the time of antibody measurement, as approved by the institutional review boards. All serum samples and clinical data had been collected prospectively before antibody analysis.

## RESULTS

### Patient characteristics

A total of 183 patients with moderately to severely active UC who initiated ADT were enrolled. Among them, 13 patients without colonoscopy data within 6 months before enrolment, 3 patients with a history of total colectomy, and 23 patients with missing baseline serum samples were excluded. Thus, 144 patients were included in the analysis. Using a cutoff value of 1.64 U/mL^6^, 142 of 144 patients (98.6%) were seropositive for anti-αvβ6 autoantibodies.

Baseline characteristics are summarized in Table 1. The median disease duration was 59.5 months, and 121 patients (84.0%) had extensive colitis. Eighty-seven patients (60.4%) were ADT-experienced. Colonoscopy was performed a median of 12.5 days before ADT initiation, and 95 patients (66.0%) had an MES of 3. According to the modified Mayo score, 66 patients (45.8%) had moderate disease. The initiated ADT included anti-TNF-α agents (n = 44 [30.6%]), vedolizumab (n = 37 [26.7%]), IL-12/23 or IL-23 inhibitors (n = 30 [20.8%]), and JAK inhibitors (n = 33 [22.9%]).

**Table 1.** Patient characteristics at baseline

	Total	Anti-αvβ6		*P*-value
		Low-level	High-level	
	n = 144	n = 103	n = 41	
Age (median in years, IQR)	41 (31–50)	40 (31–52)	41 (32–48)	0.972
Male (n, %)	89 (61.8)	59 (57.3)	30 (73.2)	0.077
Cohort				0.358
Japan (n, %)	132 (91.7)	95 (92.2)	39 (95.1)	
Israel (n, %)	12 (8.3)	10 (9.7)	2 (4.9)	
Disease duration (median in months, IQR)	59.5 (25.3–170.5)	81.0 (38.0–201.0)	31.0 (15.5–108.0)	0.004
Disease extent				
Proctitis (n, %)	1 (0.7)	1 (1.0)	0	0.527
Left-sided colitis (n, %)	22 (15.3)	18 (17.5)	4 (9.8)	0.245
Extensive colitis (n, %)	121 (84.0)	84 (81.5)	37 (90.2)	0.199
Current smoker (n, %)	21 (14.6)	14 (13.6)	7 (17.1)	0.593
Advanced therapy-experienced (n, %)	87 (60.4)	65 (63.1)	22 (53.7)	0.298
Type of prior advanced therapies used				
Anti-TNF-α agents (n, %)	66 (45.8)	49 (47.6)	17 (41.5)	0.507
Vedolizumab (n, %)	39 (27.1)	28 (27.2)	11 (26.8)	0.966
IL-12/23 or IL-23 inhibitors (n, %)	33 (22.9)	22 (21.4)	11 (26.8)	0.486
JAK inhibitors (n, %)	31 (21.5)	20 (19.4)	11 (26.8)	0.329
PRO-2 (median, IQR)	4 (3–5)	4 (3–5)	4 (3–5)	0.321
Mayo endoscopic subscore				0.235
2 (n, %)	49 (34.0)	32 (31.1)	17 (41.5)	
3 (n, %)	95 (66.0)	71 (68.9)	24 (58.5)	
Modified Mayo score				0.301
Moderate; 4–6 (n, %)	66 (45.8)	50 (48.5)	16 (39.0)	
Severe; 7–9 (n, %)	78 (54.2)	53 (51.5)	25 (61.0)	
CRP (median in mg/dL, IQR)	0.8 (0.2–2.4)	0.8 (0.2–2.1)	1.2 (0.2–2.9)	0.238
LRG (median in μg/mL, IQR)	26.0 (17.8–37.1)	22.0 (17.2–36.1)	31.5 (26.2–38.8)	0.021
FC (median in μg/g, IQR)	1,000 (466–2,911)	1,041 (410–2,908)	897 (527–3,615)	0.850
Concomitant medications				
5-aminosalicylic acid (n, %)	114 (79.2)	83 (80.6)	31 (75.6)	0.507
Topical formulations (n, %)	59 (40.9)	43 (41.8)	16 (39.0)	0.764
Immunomodulators (n, %)	44 (30.8)	29 (28.2)	15 (37.5)	0.277
Prednisolone (n, %)	41 (28.4)	29 (28.2)	12 (29.3)	0.265
Tacrolimus (n, %)	9 (6.3)	9 (8.7)	0 (0)	0.051
Initiated advanced therapies				
Anti-TNF-α agents (n, %)	44 (30.6)	27 (26.2)	17 (41.5)	0.077
Vedolizumab (n, %)	37 (26.7)	30 (29.1)	7 (17.1)	0.135
IL-12/23 or IL-23 inhibitors (n, %)	30 (20.8)	24 (23.5)	6 (14.6)	0.248
JAK inhibitors (n, %)	33 (22.9)	22 (21.4)	11 (26.8)	0.481

Patients were stratified by anti-αvβ6 autoantibody levels based on an optimal cutoff (154 U/mL) determined by the Youden index from the ROC curve assessing treatment persistence.

LRG data were missing for 32 participants (22.2%), and FC data were missing for 98 participants (68.1%).

The modified Mayo score was calculated as the sum of the Mayo stool frequency, rectal bleeding, and endoscopic subscores, with a maximum total of 9.

Statistical significance was assessed using the χ^2^ test and the Mann–Whitney *U* test.

CRP, C-reactive protein; FC, fecal calprotectin; IL, interleukin; JAK, janus kinase; LRG, leucine-rich alpha-2 glycoprotein; PRO-2, patient-reported outcome-2; ROC, receiver operating characteristic; TNF-α, tumor necrosis factor-α.

### Treatment persistence and anti-αvβ6 autoantibodies

**Figure 1.**
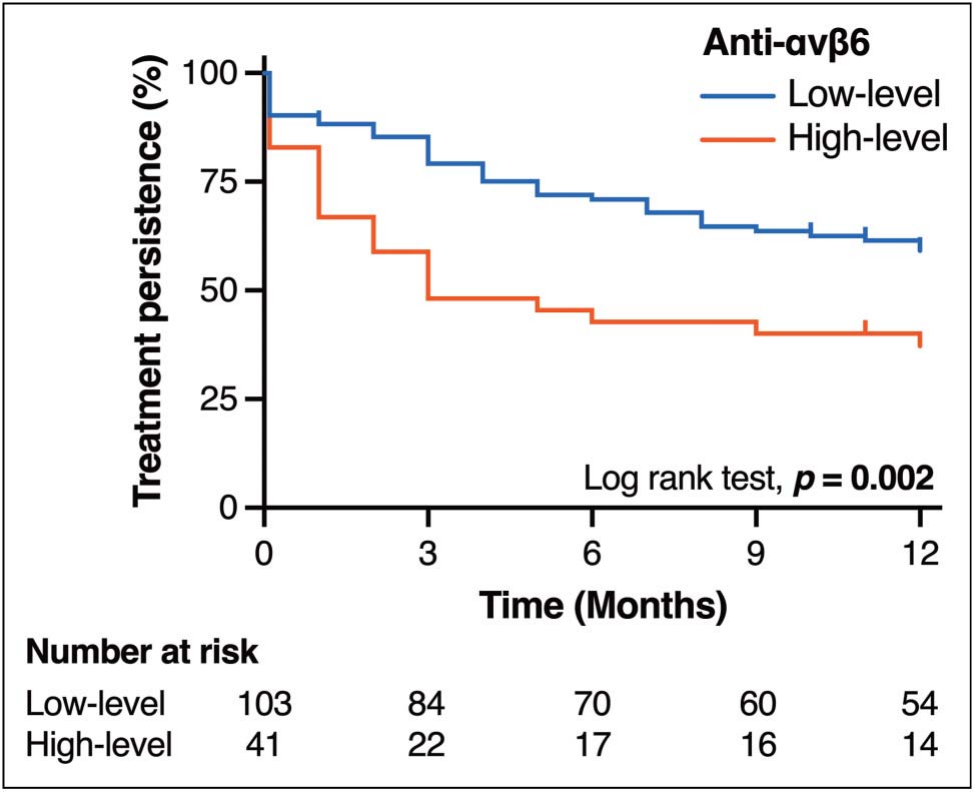
Kaplan–Meier curves for treatment persistence stratified by anti-αvβ6 autoantibody levels (low: <154 U/mL; high: ≥154 U/mL). Statistical comparison was performed using the log-rank test.

First, we examined the primary outcome, the association between anti-αvβ6 autoantibody levels and treatment persistence. The overall median observation period was 10 months, during which treatment discontinuation occurred in 70 patients (switch to another ADT because of inadequate response, n = 65; UC-related surgery, n = 5). Using the Youden index on the ROC curve, we identified an optimal cutoff value of 154 U/mL for predicting treatment persistence up to 1 year. Based on this cutoff, patients were classified into 2 groups. Anti-αvβ6 autoantibody levels were associated with disease duration and LRG, whereas MES and the modified Mayo score did not differ (Table 1). The low-level group had significantly higher treatment persistence compared with the high-level group (*P* = 0.002; Figure 1). In multivariable Cox proportional hazards analysis, anti-αvβ6 autoantibody level was the only predictor of treatment persistence (hazard ratio, 1.90; 95% confidence interval [CI], 1.09–3.32; *P* = 0.023; Table 2), independent of disease duration, disease extent, history of ADT use, the modified Mayo score, and CRP.

**Table 2.** Cox proportional hazards model analysis for predicting treatment persistence

	Crude analyses	*P-*value	HR (95% CI)	Multivariable adjusted analyses
	HR (95% CI)			*P-*value
Anti-αvβ6 (U/mL)				
High-level (≥154)	1.00 (reference)		1.00 (reference)	
Low-level (<154)	2.00 (1.20–3.33)	**0.008**	1.90 (1.09–3.32)	**0.023**
Disease duration (yr)				
≤3	1.00 (reference)		1.00 (reference)	
>3	1.18 (0.71–1.96)	0.529	0.93 (0.54–1.62)	0.818
Disease extent				
Proctitis or left-sided colitis	1.00 (reference)		1.00 (reference)	
Extensive colitis	0.67 (0.32–1.41)	0.290	0.79 (0.37–1.69)	0.550
History of advanced therapy use				
Advanced therapy-naïve	1.00 (reference)		1.00 (reference)	
Advanced therapy-experienced	1.05 (0.64–1.73)	0.832	0.96 (0.57–1.62)	0.887
Modified Mayo score				
Moderate (4–6)	1.00 (reference)		1.00 (reference)	
Severe (7–9)	0.64 (0.38–1.05)	0.077	0.68 (0.40–1.15)	0.153
CRP (per 1 mg/dL increase)	0.97 (0.91–1.05)	0.452	0.93 (0.21–5.30)	0.929

The multivariable Cox proportional hazards model included 5 potential confounders: disease duration, disease extent, history of advanced therapy use, modified Mayo score, and CRP.

The modified Mayo score was calculated as the sum of the Mayo stool frequency, rectal bleeding, and endoscopic subscores, with a maximum total of 9.

CI, confidence interval; CRP, C-reactive protein; HR, hazard ratio.

### Clinical remission rate and anti-αvβ6 autoantibodies

**Figure 2.**
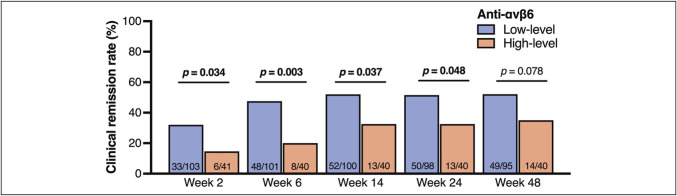
Clinical remission rates at weeks 2, 6, 14, 24, and 48 stratified by anti-αvβ6 autoantibody levels (low: <154 U/mL; high: ≥154 U/mL). At week 6, 2 patients in the low-level group and 1 in the high-level group were excluded because of intolerance. At week 14, 1 patient in the low-level group was excluded because of intolerance. At week 24, 2 patients in the low-level group were excluded because of interruption of hospital visits. At week 48, 2 patients in the low-level group were excluded because of intolerance, and 1 patient in the low-level group was excluded because of interruption of hospital visits. Statistical comparisons were performed using the χ^2^ test.

The Kaplan–Meier curves (Figure 1) suggested that the difference in treatment persistence between groups might be attributable to differences in short-term clinical response. As expected, the low-level group demonstrated significantly higher clinical remission rates than the high-level group at weeks 2, 6, 14, and 24 (Figure 2), with the most pronounced difference observed at week 6 (47.5% vs 20.0%; *P* = 0.003). Multivariable logistic regression analysis identified anti-αvβ6 autoantibody level as the only independent predictor of clinical remission at week 6 (odds ratio, 3.62; 95% CI, 1.48–9.82; *P* = 0.004; Table 3). Notably, this association remained consistent from week 2 through week 48 (see Supplementary Tables 1–4, Supplementary Digital Content, http://links.lww.com/CTG/B469). When analyzed by mechanisms of action of ADT, the differences in both treatment persistence and clinical remission rates were more evident in patients treated with JAK inhibitors (see Supplementary Figures 1A–1H, Supplementary Digital Content, http://links.lww.com/CTG/B467).

**Table 3.** Logistic regression analysis for predicting clinical remission at week 6

	Crude analyses		Multivariable adjusted analyses	
	OR (95% CI)	*P-*value	OR (95% CI)	*P-*value
Anti-αvβ6 (U/mL)				
High-level (≥154)	1.00 (reference)		1.00 (reference)	
Low-level (<154)	3.62 (1.58–9.14)	**0.002**	3.62 (1.48–9.82)	**0.004**
Disease duration (yr)				
≤3	1.00 (reference)		1.00 (reference)	
>3	1.58 (0.78–3.33)	0.208	1.18 (0.52–2.71)	0.690
Disease extent				
Proctitis or left-sided colitis	1.00 (reference)		1.00 (reference)	
Extensive colitis	0.83 (0.34–2.09)	0.688	1.01 (0.40–2.65)	0.977
History of advanced therapy use				
Advanced therapy-naïve	1.00 (reference)		1.00 (reference)	
Advanced therapy-experienced	1.08 (0.54–2.17)	0.823	1.09 (0.52–2.32)	0.818
Modified Mayo score				
Moderate (4–6)	1.00 (reference)		1.00 (reference)	
Severe (7–9)	0.64 (0.32–1.25)	0.191	0.55 (0.26–1.16)	0.117
CRP (per 1 mg/dL increase)	0.95 (0.86–1.05)	0.328	0.90 (0.80–1.20)	0.072

The multivariable Cox proportional hazards model included five potential confounders: disease duration, disease extent, history of advanced therapy use, modified Mayo score, and CRP.

The modified Mayo score was calculated as the sum of the Mayo stool frequency, rectal bleeding, and endoscopic subscores, with a maximum total of 9.

CI, confidence interval; CRP, C-reactive protein; OR, odds ratio.

### anti-αvβ6 autoantibodies and disease severity

**Figure 3.**
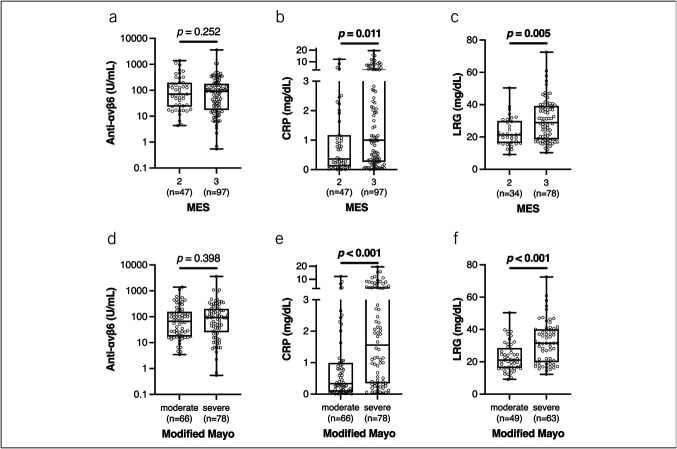
(A) Anti-αvβ6 autoantibody levels, (B) CRP levels, and (C) LRG levels, stratified by MES. (D) Anti-αvβ6 autoantibody levels, (E) CRP levels, and (F) LRG levels, stratified by the modified Mayo score (moderate: 4–6; severe: 7–9). LRG data were missing for 32 participants (22.2%). In all boxplots, the box represents the interquartile range, the center line indicates the median, and the whiskers denote the minimum and maximum values. Statistical significance was assessed using the Mann–Whitney U test. CRP, C-reactive protein; LRG, leucine-rich alpha-2 glycoprotein; MES, Mayo endoscopic subscore.

In addition, we assessed whether anti-αvβ6 autoantibody levels could serve as a biomarker of disease activity by comparing them with established serum markers such as CRP and LRG. Anti-αvβ6 autoantibody levels were not associated with endoscopic disease severity, whereas both CRP and LRG levels were significantly higher in patients with an MES of 3 compared with those with an MES of 2 (Figures 3A–3C). A similar trend was observed when disease activity was assessed using the modified Mayo score: CRP and LRG levels, but not anti-αvβ6 autoantibody levels, were significantly higher in the severe group than in the moderate group (Figures 3D–3F). Moreover, anti-αvβ6 autoantibody levels showed poor correlation with both CRP and LRG levels (see Supplementary Figures 2A and 2B, Supplementary Digital Content, http://links.lww.com/CTG/B468), while CRP and LRG exhibited a strong positive correlation with each other (see Supplementary Figure 2C, Supplementary Digital Content, http://links.lww.com/CTG/B468). These findings suggest that anti-αvβ6 autoantibodies are less reliable than CRP or LRG in reflecting disease activity, particularly in patients with moderately to severely active UC.

## DISCUSSION

This is the first study to investigate the association between anti-αvβ6 autoantibody levels and treatment outcomes of ADT in UC. Most importantly, our findings demonstrate that anti-αvβ6 autoantibodies are independent predictors of both treatment persistence and clinical remission. Furthermore, our baseline analysis indicates that anti-αvβ6 autoantibody levels are less reliable than CRP or LRG as biomarkers of disease activity in patients with moderately to severely active disease.

Integrin αvβ6 is a heterodimeric molecule expressed on colonic epithelial cells, functioning as a receptor for extracellular matrix proteins, such as fibronectin in the basement membrane (7). Several studies have reported that anti-αvβ6 autoantibody levels increase in patients with UC, whereas they are not increased in those with other intestinal diseases, such as Crohn disease, colorectal cancer, or irritable bowel syndrome, suggesting its potential as a novel diagnostic marker (4,6,7,9).

Currently, there is no standard strategy to predict response to ADT before its initiation. Only a limited number of clinical factors—such as disease severity, elevated CRP, extensive colitis, and prior exposure to anti-TNF agents—have been reported to be associated with poor response to ADT (16–18). A few biomarkers are reported to serve as a potential predictor of response. Oncostatin M, a member of the IL-6 cytokine family, has been shown to be elevated in the colonic mucosa of patients with poor prognosis and to predict primary nonresponse to both anti-TNF agents and vedolizumab (19,20). However, serum Oncostatin M levels did not predict response, indicating that local mucosal expression may be more informative for stratifying treatment outcomes (19). Regarding triggering receptor expressed on myeloid cells 1* (TREM-1)*, Verstockt et al initially reported in 2019 that low whole blood *TREM-1* expression predicted endoscopic remission after anti-TNF therapy, raising the possibility that *TREM-1* could be a predictive biomarker (21). However, a subsequent post hoc analysis of the phase 3 SERENE-UC and SERENE-CD trials failed to validate its predictive utility for clinical or endoscopic outcomes in patients treated with adalimumab, highlighting the need for further investigation before *TREM-1* can be integrated into clinical decision-making (22). Thus, at present, there is no standard predictive biomarker for response to ADT.

Livanos et al reported that anti-αvβ6 autoantibody levels at diagnosis were associated with adverse outcomes, including escalation to biologic therapy (9). Similarly, studies from Swedish and Norwegian inception cohorts demonstrated associations with aggressive disease courses (8). These findings suggest that anti-αvβ6 autoantibodies may serve as a prognostic marker for poor outcomes. However, since antibody levels were measured at the time of diagnosis, they may simply reflect baseline disease severity, leading to poor prognosis. However, our data showed no correlation between anti-αvβ6 autoantibody levels and disease activity, as measured by MES and the modified Mayo score. In addition, extensive disease, elevated CRP, prior biologic exposure, and short disease duration (≦3 years) have also been reported to be associated with worse treatment persistence of ADT (23–25). In our study, anti-αvβ6 autoantibodies remained the only independent predictor of treatment persistence after adjusting for these variables. Thus, anti-αvβ6 autoantibodies may function not only as a prognostic marker at diagnosis but also as a predictor of long-term treatment outcomes after the initiation of ADT.

In addition to treatment persistence, the present study showed that low anti-αvβ6 autoantibody levels were consistently associated with higher clinical remission rates from weeks 2–24, with the strongest effect at week 6, where remission was nearly 4 times more likely. A consensus statement in Selecting Therapeutic Targets in Inflammatory Bowel Disease-II (STRIDE-II) has highlighted clinical remission as a crucial short-term treatment target for UC therapy (26). Our results suggest that anti-αvβ6 autoantibodies may serve as a novel biomarker for predicting short-term response to ADT in UC. In fact, such short-term response in patients with low-antibody levels leads to longer-term remission and treatment persistence over a year.

Taken together, these findings indicate that high anti-αvβ6 autoantibody levels may represent a “signature” of the difficult-to-treat UC phenotype (27). Although the sample size was insufficient to draw definitive conclusions for each mechanism of action, patients in the high-level group showed particularly low response to JAK inhibitors. Further investigation in larger cohorts is warranted. For these difficult-to-treat patients, early introduction of the most potent therapies, dose escalation, advanced combination therapy, or even surgical intervention may be necessary to achieve optimal disease control (28).

The strength of this study lies in its prospective evaluation of clinical effectiveness at predefined timepoints, with baseline anti-αvβ6 autoantibody levels measured later in a blinded manner, ensuring unbiased analysis. Moreover, multivariable analysis incorporating known prognostic factors confirmed anti-αvβ6 autoantibody level as an independent predictor of treatment outcomes.

However, several limitations should be acknowledged. In addition to the insufficient sample size for subgroup analyses by mechanism of action, this was an observational study, which may have caused unmeasured confounding factors affecting treatment persistence. Furthermore, outcome assessments were based on clinical symptoms and treatment continuity, while objective markers such as fecal calprotectin and endoscopic findings were not uniformly collected, potentially limiting the accuracy of disease activity evaluation.

In conclusion, although anti-αvβ6 autoantibody levels did not correlate with conventional disease activity indices, they effectively predicted treatment outcomes after the initiation of ADT in patients with moderately to severely active UC. These findings highlight their potential as a biomarker for personalized treatment strategies. Further research is warranted to clarify the relevance of anti-αvβ6 autoantibodies in clinical practice.

## CONFLICTS OF INTEREST

**Guarantor of the article:** Taku Kobayashi, MD, PhD.

**Specific author contributions:** S.S. led the conceptualization, methodology, investigation, formal analysis, data curation, visualization, and drafting of the manuscript, and contributed equally to validation and critical revision. K.A. contributed equally to conceptualization and methodology, led validation, and supported drafting and critical revision of the manuscript. S.K. and S.O. contributed to investigation, formal analysis, and manuscript revision. G.B. contributed to the investigation and critically reviewed the manuscript. N.M. and T.K. supervised the study, with T.K. leading critical review and editing of the manuscript. All authors reviewed the manuscript critically for important intellectual content and approved the final version.

**Financial support:** None to report.

**Potential competing interests:** Shunsuke Shibui has served as a speaker for AbbVie. Shinji Okabayashi has received speaking fees from Mitsubishi Tanabe Pharma and Mochida Pharmaceutical, and consulting fees from EA Pharma. Shintaro Sagami has served as an advisory board member or speaker for AbbVie, Alimentiv, Eli Lilly, Janssen Pharmaceutical K.K., Gilead Sciences, Inc., JIMRO Co., Ltd., KISSEI Pharmaceutical Co., Ltd., Kyorin Pharmaceutical Co., Ltd., Mitsubishi Tanabe Pharma Corporation, EA Pharma Co., Takeda Pharmaceutical Co., Ltd., Nippon Kayaku Co., Ltd., and Zeria Pharmaceutical Co., Ltd. and has received research grants from Gilead Sciences, Bristol-Myers Squibb, and Ferring Pharmaceuticals. Masaru Nakano has served as a speaker or a consultant in Covidien, Mochida Pharmaceutical, Takeda Pharmaceutical, Zeria Pharmaceutical, Kyorin Pharmaceutical, and Nippon Kayaku; received research funding from Mitsubishi Tanabe Pharma and the Japanese foundation for research and promotion of endoscopy. Toshifumi Hibi has received lecture fees from, Abbvie GK, JIMRO, Mitsubishi-Tanabe Pharma, Mochida Pharmaceutical, Sand K.K., Takeda Pharmaceutical, Zeria Pharmaceutical Co., Ltd., Advisory/consultancy fees from Abbvie GK, Celltrion, Eli Lilly, Gilead Sciences, Mitsubishi-Tanabe Pharma., Takeda Pharmaceutical, Zeria Pharmaceutical and research grants from AbbvieGK, Activaid, Alfresa Pharma Corporation, JMDC Inc., Gilead Sciences, Inc., Nippon Kayaku Co., Ltd., Eli Lilly Japan K.K., Mochida Pharmaceutical Co., Ltd., Janssen Pharmaceutical K.K., Pfizer Japan Inc., Takeda Pharmaceutical Co., Ltd., Ferring Pharmaceuticals and Bristol-Myers Squibb; belonged to study group sponsorship by Alfresa Pharma Corporation, JIMRO Co., Ltd., Kyorin Pharmaceutical Co., Ltd., and Mochida Pharmaceutical Co., Ltd. Miyarisan Pharmaceutical Co., Ltd. Zeria Pharmaceutical Co., Ltd. Nitsan Maharshak has received speaking and consulting fees from Pfizer, Abbvie, Lilly, Takeda, Janssen, Ferring, BiomX, BMS, Nestle, Teva, and grant support from Takeda, Janssen, Abbott, Abbvie, Pfizer, BMS, Corundum Innovation Ltd, Nestle and from the Helmsely Charitable Trust. Shin Maeda has received lecture fees from Abbvie, Mitsubishi-Tanabe Pharma, Mochida Pharmaceutical, Takeda Pharmaceutical, Zeria Pharmaceutical and EA Pharma, and research expenses from Otsuka Pharmaceutical, Chugai Pharmaceutical, Mochida pharmaceutical, Zeria Pharmaceutical, and Kyowa-Kirin. Taku Kobayashi served as an advisory board member, consultant, or speaker for AbbVie, Alfresa Pharma, Alimentiv, Bristol Myers Squibb, Celltrion, Covidien, EA Pharma, Eli Lilly, Ferring Pharmaceuticals, Galapagos, Gilead Sciences, Janssen Pharmaceuticals, JIMRO, Kissei Pharmaceutical, Kyorin Pharmaceutical, Mitsubishi Tanabe Pharma, Mochida Pharmaceutical, MSD, Nippon Kayaku, Pfizer, Takeda, and Zeria Pharmaceutical, and has received research funding from AbbVie, Alfresa Pharma, Bristol Myers Squibb, EA Pharma, Gilead Sciences, Kyorin Pharmaceutical, Mochida Pharmaceutical, Nippon Kayaku, Otsuka Holdings, Pfizer, Sekisui Medical, Samsung, Takeda, and Zeria Pharmaceutical.

**Data Transparency Statement:** The data supporting the findings of this study are available on request from the corresponding authors. The data are not publicly available because of privacy and ethical restrictions.

**Study Highlights**

**WHAT IS KNOWN**

✓ Reliable biomarkers to predict individual responses to advanced therapies in UC are currently lacking.
✓ Anti-αvβ6 autoantibodies serve as a specific diagnostic biomarker and are associated with poor prognosis in UC.

**WHAT IS NEW HERE**

✓ Low anti-αvβ6 levels independently predict superior one-year treatment persistence and clinical remission.
✓ Anti-αvβ6 levels do not reflect current disease severity, unlike CRP or LRG.
✓ This biomarker identifies difficult-to-treat subtypes, guiding personalized treatment and precision medicine.

## Supplementary Material

**Figure:**
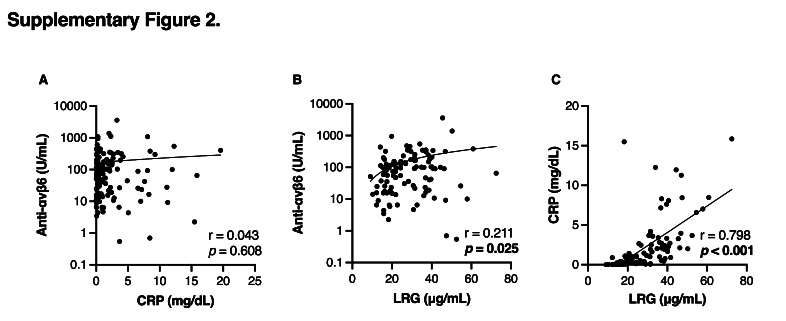


**Figure:**
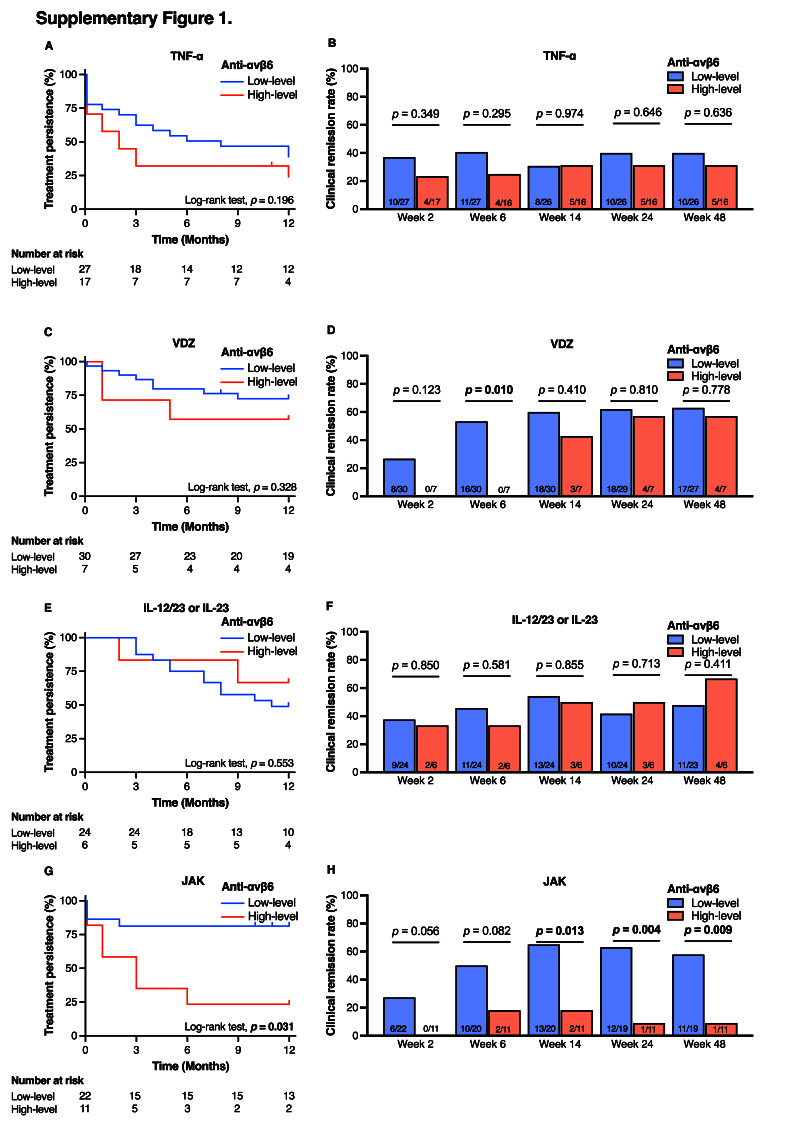


## ABBREVIATIONS:

ADTs: advanced therapies
CRP: C-reactive protein
HR: hazard ratio
IL: interleukin
IQR: interquartile range
JAK: Janus kinase
LRG: Leucine-rich alpha-2 glycoprotein
MES: Mayo endoscopic subscore
PRO-2: patient-reported outcome-2
ROC: receiver-operating characteristic
STRIDE: the selecting therapeutic targets in inflammatory bowel disease
TNF-α: anti-integrin αvβ6
anti-αvβ6: tumor necrosis factor-α
*TREM-1*: *Triggering Receptor Expressed on Myeloid cells 1*
UC: ulcerative colitis
VDZ: vedolizumab
