# Supplementary material for: Anti-Integrin αvβ6 Autoantibodies Predict Response and Treatment Persistence to Advanced Therapies in Ulcerative Colitis
Source: Clin Transl Gastroenterol. 2026 Feb 3;17(3):e00990. doi: 10.14309/ctg.0000000000000990 (PMC13008197; doi:10.14309/ctg.0000000000000990)
Supplement: Supplementary file 1 [file ct9-17-e00990-s001.docx]

【**Figure Legends**】

**Supplementary Figure 1.**

(A, C, E, G) Kaplan–Meier curves for treatment persistence and (B, D, F, H) clinical remission rates at weeks 2, 6, 14, 24, and 48, stratified by anti-αvβ6 autoantibody levels (low: <154 U/mL; high: ≥154 U/mL), are shown across treatment groups: (A, B) anti-tumor necrosis factor-α (TNF-α) agents, (C, D) vedolizumab (VDZ), (E, F) Interleukin (IL)-12/23 or IL-23 inhibitors, and (G, H) Janus kinase (JAK) inhibitors. In the TNF-α group, one patient in the high-level group was excluded at week 6 due to intolerance, and one patient in the low-level group was excluded at week 14 for the same reason. In the VDZ group, one patient in the low-level group was excluded at week 24 due to interruption of hospital visits, and at week 48, two patients in the low-level group were excluded due to intolerance. In the IL-12/23 or IL-23 group, one patient in the low-level group was excluded due to intolerance. In the JAK inhibitors group, two patients in the low-level group were excluded at week 6 due to intolerance, and one patient in the low-level group was excluded at week 24 due to interruption of hospital visits. Statistical comparisons were performed using the log-rank test and the chi-square test.

**Supplementary Figure 2.**

(A) Spearman correlation between anti-αvβ6 autoantibody levels and C-reactive protein (CRP) levels; (B) anti-αvβ6 autoantibody levels and leucine-rich alpha-2 glycoprotein (LRG) levels; and (C) CRP levels and LRG levels. LRG data were missing for 32 participants (22.2%). Statistical significance was assessed using the Spearman correlation.

**【Tables】**

| **Supplementary Table 1.** Logistic regression analysis for predicting clinical remission at week 2. | | | | | |
| --- | --- | --- | --- | --- | --- |
|  | **Crude analyses** | |  | **Multivariable adjusted analyses** | |
|  | **OR (95% CI)** | ***p-*value** |  | **OR (95% CI)** | ***p-*value** |
| Anti-αvβ6 |  |  |  |  |  |
| High-level (≧154U/mL) | 1.00 (reference) |  |  | 1.00 (reference) |  |
| Low-level (<154U/mL) | 2.75 (1.11–7.83) | **0.027** |  | 2.99 (1.08–9.62) | **0.034** |
| Disease duration |  |  |  |  |  |
| ≦ 3 years | 1.00 (reference) |  |  | 1.00 (reference) |  |
| > 3 years | 1.44 (0.66–3.33) | 0.364 |  | 1.29 (0.51–3.41) | 0.596 |
| Disease extent |  |  |  |  |  |
| Proctitis or left-sided colitis | 1.00 (reference) |  |  | 1.00 (reference) |  |
| Extensive colitis | 1.41 (0.51–4.53) | 0.521 |  | 1.80 (0.62–6.16) | 0.289 |
| History of advanced therapy use |  |  |  |  |  |
| Advanced therapy-naïve | 1.00 (reference) |  |  | 1.00 (reference) |  |
| Advanced therapy-experienced | 0.92 (0.44–1.97) | 0.829 |  | 0.98 (0.43–2.29) | 0.968 |
| Modified Mayo score |  |  |  |  |  |
| Moderate (4–6) | 1.00 (reference) |  |  | 1.00 (reference) |  |
| Severe (7–9) | 0.64 (0.30–1.34) | 0.240 |  | 0.42 (0.17–0.97) | **0.042** |
| CRP (per 1mg/dL increase) | 0.88 (0.79–0.97) | **0.013** |  | 0.83 (0.73–1.21) | **0.001** |
| The multivariable Cox proportional hazards model included five potential confounders: disease duration, disease extent, history of advanced therapy use, modified Mayo score, and CRP. The modified Mayo score was calculated as the sum of the Mayo stool frequency, rectal bleeding, and endoscopic subscores, with a maximum total of 9.  Abbreviations: CI, confidence interval; CRP, C-reactive protein; OR, odds ratio. | | | | | |

| **Supplementary Table 2.** Logistic regression analysis for predicting clinical remission at week 14. | | | | | |
| --- | --- | --- | --- | --- | --- |
|  | **Crude analyses** | |  | **Multivariable adjusted analyses** | |
|  | **OR (95% CI)** | ***p-*value** |  | **OR (95% CI)** | ***p-*value** |
| Anti-αvβ6 |  |  |  |  |  |
| High-level (≧154U/mL) | 1.00 (reference) |  |  | 1.00 (reference) |  |
| Low-level (<154U/mL) | 2.25 (1.06–4.97) | **0.035** |  | 2.91 (1.26–7.12) | **0.012** |
| Disease duration |  |  |  |  |  |
| ≦ 3 years | 1.00 (reference) |  |  | 1.00 (reference) |  |
| > 3 years | 0.91 (0.45–1.84) | 0.799 |  | 0.65 (0.29–1.44) | 0.292 |
| Disease extent |  |  |  |  |  |
| Proctitis or left-sided colitis | 1.00 (reference) |  |  | 1.00 (reference) |  |
| Extensive colitis | 1.43 (0.58–3.67) | 0.441 |  | 1.82 (0.72–4.82) | 0.210 |
| History of advanced therapy use |  |  |  |  |  |
| Advanced therapy-naïve | 1.00 (reference) |  |  | 1.00 (reference) |  |
| Advanced therapy-experienced | 0.83 (0.42–1.64) | 0.596 |  | 0.85 (0.41–1.77) | 0.671 |
| Modified Mayo score |  |  |  |  |  |
| Moderate (4–6) | 1.00 (reference) |  |  | 1.00 (reference) |  |
| Severe (7–9) | 0.76 (0.38–1.48) | 0.424 |  | 0.71 (0.34–1.47) | 0.362 |
| CRP (per 1mg/dL increase) | 0.98 (0.87–1.09) | 0.646 |  | 0.95 (0.84–1.07) | 0.375 |
| The multivariable Cox proportional hazards model included five potential confounders: disease duration, disease extent, history of advanced therapy use, modified Mayo score, and CRP. The modified Mayo score was calculated as the sum of the Mayo stool frequency, rectal bleeding, and endoscopic subscores, with a maximum total of 9.  Abbreviations: CI, confidence interval; CRP, C-reactive protein; OR, odds ratio. | | | | | |

| **Supplementary Table 3.** Logistic regression analysis for predicting clinical remission at week 24. | | | | | |
| --- | --- | --- | --- | --- | --- |
|  | **Crude analyses** | |  | **Multivariable adjusted analyses** | |
|  | **OR (95% CI)** | ***p-*value** |  | **OR (95% CI)** | ***p-*value** |
| Anti-αvβ6 |  |  |  |  |  |
| High-level (≧154U/mL) | 1.00 (reference) |  |  | 1.00 (reference) |  |
| Low-level (<154U/mL) | 2.16 (1.02–4.80) | **0.046** |  | 2.64 (1.15–6.41) | **0.022** |
| Disease duration |  |  |  |  |  |
| ≦ 3 years | 1.00 (reference) |  |  | 1.00 (reference) |  |
| > 3 years | 0.87 (0.43–1.76) | 0.697 |  | 0.63 (0.28–1.38) | 0.247 |
| Disease extent |  |  |  |  |  |
| Proctitis or left-sided colitis | 1.00 (reference) |  |  | 1.00 (reference) |  |
| Extensive colitis | 1.01 (0.40–2.57) | 0.984 |  | 1.24 (0.48–3.26) | 0.654 |
| History of advanced therapy use |  |  |  |  |  |
| Advanced therapy-naïve | 1.00 (reference) |  |  | 1.00 (reference) |  |
| Advanced therapy-experienced | 0.89 (0.45–1.76) | 0.734 |  | 0.94 (0.46–1.95) | 0.874 |
| Modified Mayo score |  |  |  |  |  |
| Moderate (4–6) | 1.00 (reference) |  |  | 1.00 (reference) |  |
| Severe (7–9) | 0.64 (0.32–1.26) | 0.195 |  | 0.61 (0.29–1.26) | 0.185 |
| CRP (per 1mg/dL increase) | 0.99 (0.89–1.11) | 0.908 |  | 0.96 (0.85–1.09) | 0.536 |
| The multivariable Cox proportional hazards model included five potential confounders: disease duration, disease extent, history of advanced therapy use, modified Mayo score, and CRP. The modified Mayo score was calculated as the sum of the Mayo stool frequency, rectal bleeding, and endoscopic subscores, with a maximum total of 9.  Abbreviations: CI, confidence interval; CRP, C-reactive protein; OR, odds ratio. | | | | | |

| **Supplementary Table 4.** Logistic regression analysis for predicting clinical remission at week 48. | | | | | |
| --- | --- | --- | --- | --- | --- |
|  | **Crude analyses** | |  | **Multivariable adjusted analyses** | |
|  | **OR (95% CI)** | ***p-*value** |  | **OR (95% CI)** | ***p-*value** |
| Anti-αvβ6 |  |  |  |  |  |
| High-level (≧154U/mL) | 1.00 (reference) |  |  | 1.00 (reference) |  |
| Low-level (<154U/mL) | 1.98 (0.93–4.33) | 0.076 |  | 2.38 (1.04–5.76) | **0.041** |
| Disease duration |  |  |  |  |  |
| ≦ 3 years | 1.00 (reference) |  |  | 1.00 (reference) |  |
| > 3 years | 0.82 (0.40–1.67) | 0.577 |  | 0.63 (0.28–1.39) | 0.253 |
| Disease extent |  |  |  |  |  |
| Proctitis or left-sided colitis | 1.00 (reference) |  |  | 1.00 (reference) |  |
| Extensive colitis | 0.60 (0.23–1.55) | 0.295 |  | 0.71 (0.37–1.86) | 0.486 |
| History of advanced therapy use |  |  |  |  |  |
| Advanced therapy-naïve | 1.00 (reference) |  |  | 1.00 (reference) |  |
| Advanced therapy-experienced | 0.75 (0.38–1.49) | 0.413 |  | 0.76 (0.37–1.58) | 0.468 |
| Modified Mayo score |  |  |  |  |  |
| Moderate (4–6) | 1.00 (reference) |  |  | 1.00 (reference) |  |
| Severe (7–9) | 0.83 (0.42–1.63) | 0.580 |  | 0.85 (0.41–1.78) | 0.668 |
| CRP (per 1mg/dL increase) | 0.99 (0.89–1.10) | 0.829 |  | 0.99 (0.87–1.11) | 0.821 |
| The multivariable Cox proportional hazards model included five potential confounders: disease duration, disease extent, history of advanced therapy use, modified Mayo score, and CRP. The modified Mayo score was calculated as the sum of the Mayo stool frequency, rectal bleeding, and endoscopic subscores, with a maximum total of 9.  Abbreviations: CI, confidence interval; CRP, C-reactive protein; OR, odds ratio. | | | | | |
